# Meta-Analysis of Efficacy From CTLA-4 and PD-1/PD-L1 Inhibitors in Cancer Patients

**DOI:** 10.3389/fonc.2022.876098

**Published:** 2022-04-28

**Authors:** Li Xu, Xin Yan, Weiyue Ding

**Affiliations:** ^1^College of Computer Science and Technology, Harbin Engineering University, Harbin, China; ^2^Key Laboratory of Symbolic Computation and Knowledge Engineering of Ministry of Education, Jilin University, Changchun, China; ^3^School of Mathematics, Harbin Institute of Technology, Harbin, China

**Keywords:** immune checkpoint inhibitors, meta-analysis, cytotoxic T-lymphocyte-associated protein 4, overall survival, programmed cell death 1, progression-free survival, programmed death ligand 1

## Abstract

**Introduction:**

Immune checkpoint inhibitors (ICIs) have been approved to prolong overall survival (OS), compared to other treatments. However, the recent studies reported consistent and inconsistent results. Hence, we conducted this meta-analysis to evaluate the efficacy of ICIs.

**Materials and Methods:**

The articles were identified by searching PubMed, Embase, and Google Scholar published up to December 2021. A total of 12,126 participants (6,450 cases and 5,676 controls) were involved in the meta-analysis. Median OS and median progression-free survival (PFS) were selected to evaluate the efficacy of cytotoxic T-lymphocyte-associated protein 4 (CTLA-4), programmed cell death 1 (PD-1), and programmed death ligand 1 (PD-L1) inhibitors (ipilimumab, nivolumab or pembrolizumab, and atezolizumab, respectively). Utilizing the random-effect model, hazard ratios (HRs) with 95 confidence intervals (CIs) were calculated by R software.

**Results:**

We observed a significant association between cancer patients and ICIs in OS (HR = 0.79, CI = 0.74–0.84) and PFS (HR = 0.80, CI = 0.75–0.86).

**Conclusions:**

The meta-analysis suggested that ICIs were associated with obvious improvements in PFS and OS compared with non-ICI therapies.

## Introduction

Cancer, an enormous burden on society, is one of the main reasons of death in both developed and developing countries. According to the global cancer statistics, there were about 19.3 million new cancer cases and nearly 10.0 million cancer deaths in 2020 worldwide (1). The immune system can recognize and prepare to eliminate cancer but is controlled by inhibitory receptors and ligands (2). Immune checkpoints are regulatory pathways in the immunome that inhibit a part of an active immune response against a specific target or a group of targets (3). These immune checkpoint pathways are often able to keep self-tolerance and limit incidental tissue damage during the antimicrobial immune response; thus, immune destruction can be averted by cancers. There is no doubt that tumors co-opt certain immune checkpoint pathways as a main mechanism of immune resistance, especially against T cells that are specific for tumor antigens (4).

Immune checkpoint inhibitors (ICIs), which regain the efficacy of tumor-specific T cells in the tumor microenvironment, enhancing the immune system’s ability to recognize and eradicate tumors, are breakthroughs in the treatment of cancer and have made significant advances in both hematological and solid tumor oncology (5). They have been approved for use in melanoma, bladder cancer, non-small cell lung cancer (NSCLC), stomach cancer, renal cell carcinoma (RCC) and head and neck squamous cell carcinoma and will be approved for other types in the foreseeable future tumors (6, 7).

The US Food and Drug Administration (FDA) approved ipilimumab as the first CTLA-4 inhibitor of advanced melanoma. Nivolumab and pembrolizumab were the first of two PD-1 inhibitors approved for advanced melanoma, and atezolizumab was the first programmed death ligand 1 (PD-L1) inhibitor approved by the FDA (8–11).

Recent studies showed that ICIs could prolong the overall survival (OS) of cancer patients, compared with placebo, dacarbazine, everolimus, paclitaxel, chemotherapy, and other therapy methods or drugs (12–24). However, the studies reported inconsistent results. In 2013, Reck et al. randomly assigned 130 SCLC patients to receive paclitaxel with placebo (control) or ipilimumab 10 mg/kg in two alternative regimens, concurrent ipilimumab or phased ipilimumab, and declared that ipilimumab did not prolong the overall survival (OS) of SCLC patients (25). In 2014, Kwon et al. did a double-blind, multicenter, randomized, phase 3 trial with 799 metastatic castration-resistant prostate cancer (399 to ipilimumab and 400 to placebo) patients and reported that no obvious difference in overall survival was found between the ipilimumab group and the placebo group (26). In 2016, Beer et al. randomly assigned 400 and 202 metastatic castration-resistant prostate cancer patients to ipilimumab and to placebo, respectively, and discovered that ipilimumab did not increase the overall survival (OS) of patients with metastatic castration-resistant prostate cancer (27). Reck et al. randomly assigned 478 small-cell lung cancer (SCLC) patients to the chemotherapy plus ipilimumab group and 476 SCLC patients to the chemotherapy plus placebo group and got a conclusion that ipilimumab plus chemotherapy did not prolong OS compared with chemotherapy alone in SCLC patients (28). Larkin et al. randomly assigned 272 melanoma patients to the nivolumab group and 133 melanoma patients to chemotherapy and found that nivolumab did not prolong OS compared with chemotherapy alone in SCLC (29). Owonikoko et al. randomly assigned 278 SCLC patients to the ipilimumab group and 278 SCLC patients to the chemotherapy group and found that ipilimumab did not prolong OS compared with chemotherapy alone in SCLC patients (30). Spigel et al. randomly assigned 284 SCLC patients to the nivolumab group and 285 SCLC patients to the chemotherapy group and got a conclusion that nivolumab did not prolong OS compared with chemotherapy alone in SCLC patients (31). Hence, to get a more convincing result, we performed a meta-analysis to study the efficacy of ipilimumab, nivolumab, pembrolizumab, and atezolizumab, compared to other therapies or other drugs.

## Materials and Methods

### Search Strategy

We identified all randomized clinical trials that compared ipilimumab, nivolumab, pembrolizumab, or atezolizumab with the non-immunotherapy control arms from January 1, 2007, to December 31, 2021. The articles we collected were searched by using the keywords “overall survival” or “OS,” “progression-free survival” or “PFS,” “immune checkpoint inhibitors” or “immune checkpoint blockage” or “ICIs” or “ipilimumab” or “nivolumab” or “pembrolizumab” or “atezolizumab” in the PubMed, Google Scholar and Embase databases. The articles we selected were written in English.

### Study Selection Criteria

Trials were eligible for inclusion if they met the following criteria: (1) trials that involved patients must receive cancer treatment; (2) trials that had adequate data available including OS and PFS; (3) trials were phase 2 or phase 3 randomized clinical trials (RCTs); and (4) the articles published must be written in English.

### Data Extraction

We extracted the following information from each study and selected the items including first author’s last name, year of publication, phase of RCTs, the name of the ICIs (ipilimumab, nivolumab, pembrolizumab or atezolizumab) and control arms, number of patients ICIs and control groups, and the hazard ratios (HRs) of OS and PFS. All the duplicated studies were excluded.

### Statistics Analysis

To calculate the overall incidence and HR of OS and PFS, we combined estimates by exploiting the fixed-effect model with the Mantel and Haenszel method and by employing the random-effect models with the DerSimonian and Laird method. The statistical analysis was performed with the R software package named Meta. The HR with 95% confidence interval (CI) was calculated to access the association between overall survival and ICIs.

Two quantities, Cochran’s Q and I^2^, were used to access the heterogeneity in different types of ICIs groups and subgroups. Statistical heterogeneity was assessed using Cochrane’s Q statistic, and the p value ranging from 0% to 100%, to measure the significance level of inconsistency. If the value of value I^2^ is less than 50%, or the p value of heterogeneity is greater than 0.10, the fixed-effect model is applied, otherwise the random effect model is employed. After the heterogeneity test, we exploited the R meta package to conduct the meta-analysis with the random-effect model.

Egger’s test (32) and Begg’s test (33) were selected to access the publication bias for OS and PFS. When a two-tailed p value was less than 0.05, the publication bias was extremely significant. Moreover, the potential publication bias was assessed by Begg’s funnel plots to check the relative symmetry of the overall estimated individual study estimates.

## Results

### Literature Search

**Figure 1:**
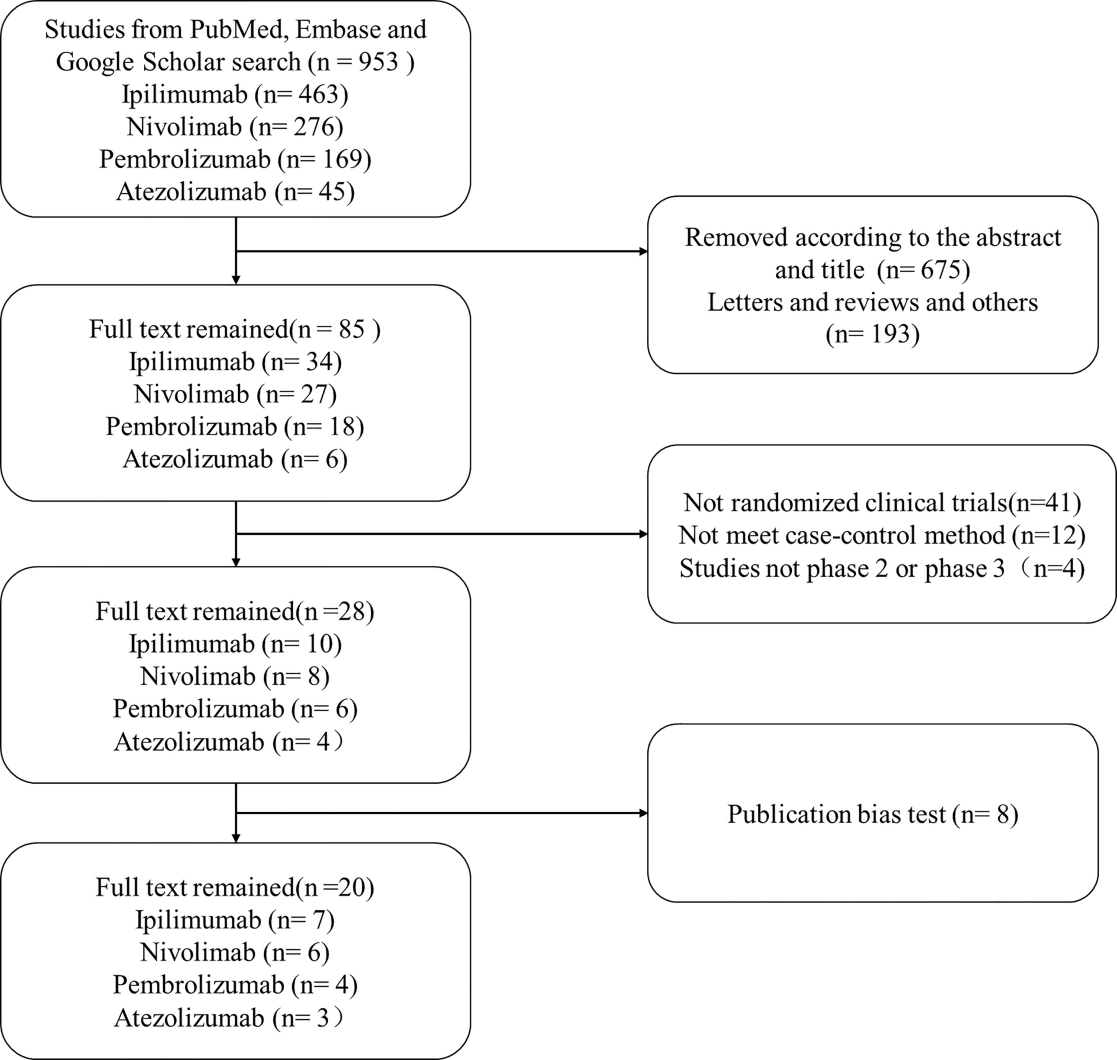
The flowchart of collecting articles. Through the search strategy, a total of 953 articles were identified. According to the title and abstract, 675 articles were primarily deleted, 193 articles were removed because they were letters, reviews, and other articles, 41 articles were excluded as they were non-randomized clinical trials, 12 articles were removed because they did not meet our case–control method, and 4 articles were deleted because studies were not phase 2 or phase 3 RCTs. Then 28 were remained. Moreover, 8 articles were removed after the publication bias test.

A flowchart for the article selection is shown in **Figure 1**. Through the search strategy, a total of 953 articles were identified. According to the title and abstract, 675 articles were primarily deleted, 193 articles were removed because they were letters, reviews, and other articles, 41 articles were excluded as they were not randomized clinical trials (RCTs), 12 articles were removed because they did not meet our case–control method, and 4 articles were deleted because studies were not phase 2 or phase 3 RCTs. Then 28 were remained, and 8 articles were removed because of the publication bias test. Finally, 20 articles were left, including 7 ipilimumab articles, 6 nivolumab articles, 4 pembrolizumab articles, and 3 atezolizumab articles, respectively (shown in **Table 1**). Moreover, the data we extracted from the articles were accessed in clinical trial databases with specific identifiers.

**Table 1:** The primary characteristics of the 23 articles.

Study	Year	Treatment	Arm	Phase	Tumor	No.		OS		PFS	
						ICIs	Control	HR	95%CI	HR	95%CI
Hodi et al. (12)	2010	Ipi+Gp100	Gp100	3	Melanoma	403	136	0.68	0.55–0.65	0.81	0.66–1
Hodi et al. (12)	2010	Ipilimumab	Gp100	3	Melanoma	137	136	0.66	0.51–0.87	0.64	0.5–0.83
Robert et al. (13)	2011	Ipi+ DTIC	Dacarbazine	3	Melanoma	250	252	0.716	0.558–0.872	NA	NA
Reck et al. (25)	2013	CP+Con Ipi Chemotherapy	Chemotherapy	2	SCLC	43	55	0.947	0.585–1.583	0.93	0.588–1.481
Reck et al. (25)	2013	CP+Seq Ipi	Chemotherapy	2	SCLC	42	55	0.753	0.461–1.232	0.927	0.59–1.45
Kwon et al. (26)	2014	Ipilimumab	Placebo	3	Prostate	399	400	0.83	0.71–0.96	0.7	0.61–0.82
Weber et al. (17)	2015	Nivolumab	Chemotherapy	3	Melanoma	272	133	0.95	0.73–1.24	1.03	0.78–1.36
Brahmer et al. (15)	2015	Nivolumab	Docetaxel	3	NSCLC	135	137	0.59	0.43–0.81	0.62	0.47–0.81
Borghaei et al. (14)	2015	Nivolumab	Docetaxel	3	NSCLC	292	290	0.73	0.59–0.89	0.92	0.77–1.11
Ribas et al. (16)	2015	Pembrolizumab	Chemotherapy	2	Melanoma	180	179	0.87	0.67–1.12	0.58	0.46–0.73
Beer et al. (27)	2016	Ipilimumab	Placebo	3	Prostate	400	202	1.11	0.88–1.39	0.67	0.55–0.8
Reck et al. (28)	2016	Ipilimumab	VP16+Plt	3	SCLC	478	476	0.936	0.807–1.085	0.85	0.75–0.97
Herbst et al. (19)	2016	Pembrolizumab 2mg	Chemotherapy	3	NSCLC	344	343	0.71	0.58–0.88	0.88	0.73–1.04
Herbst et al. (19)	2016	Pembrolizumab 10mg	Chemotherapy	3	NSCLC	346	343	0.61	0.49–0.75	0.79	0.66–0.94
Fehrenbacher et al. (18)	2016	Atezolizumab	Docetaxel	3	NSCLC	144	133	0.69	0.52–0.92	0.92	0.71–1.2
Rittmeyer et al. (21)	2016	Atezolizumab	Docetaxel	3	NSCLC	425	425	0.73	0.62–0.81	0.95	0.82–1.1
Bellmunt et al. (20)	2017	Pembrolizumab	Chemotherapy	3	Urothelial	270	272	0.73	0.59–0.91	0.98	0.81–1.19
Larkin et al. (29)	2018	Nivolumab	Chemotherapy	3	Melanoma	272	133	0.95	0.70–1.29	1	0.78–1.44
Paz-Ares et al. (22)	2019	Nivolumab + chemo	Chemotherapy	3	NSCLC	377	388	0.81	0.67–0.97	0.62	0.52–0.73
Owonikoko et al. (30)	2019	Ipilimumab	Placebo	3	SCLC	278	278	0.84	0.69–1.02	0.67	0.56–0.81
Rudin et al. (24)	2020	Pembrolizumab + etoposide	Placebo+ etoposide	3	SCLC	228	225	0.8	0.64–0.98	0.75	0.61–0.91
Galsky et al. (23)	2020	Atezolizumab + chemotherapy	Placebo+ chemotherapy	3	Urothelial	451	400	0.8	0.70–0.96	0.83	0.69–1.0
Spigel et al. (31)	2021	Nivolumab	Chemotherapy	3	SCLC	284	285	0.86	0.72–1.04	1.41	1.18–1.69

As shown in **Table 1**, a total of 12,126 participants (6,450 cases and 5,676 controls) from 20 articles were included in the meta-analysis. The name of the first author, the publication year, the tumor type of the study, the phase of the RCTs, the name of the ICIs (ipilimumab, nivolumab, pembrolizumab, or atezolizumab) in the experimental groups and non-ICI therapies in the control groups, the number of patients in the ICIs and control groups, and the HR of OS and PFS.

### Heterogeneity Test

The summary result of heterogeneity is directly shown in **Table 2**. In the OS group, we found little heterogeneity in total with *I*^2^ = 41%, *p* = 0.02, and we chose to select the random-effect model according to the method we used. In the tumor subgroup heterogeneity test, we did not find obvious significant heterogeneity in the melanoma (*I*^2^ = 38%, *p* = 0.16), SCLC (*I*^2^ = 0%, *p*=0.83), NSCLC (*I^2^* = 0%, *p*=0.48), and urothelial cancer (*I*^2^ = 0%, *p* = 0.73) subgroups, but a significant heterogeneity was found in the prostate cancer (*I*^2^ = 77%, *p* = 0.04) subgroup. In the PFS group, we also found heterogeneity in total with *I*^2^ = 58%, *p* < 0.01, and we chose to select the random-effect model according to the method we used. In the tumor subgroup heterogeneity test, we did not find obvious significant heterogeneity in the SCLC (*I*^2^ = 28%, *p* = 0.24), NSCLC (*I^2^* = 45%, *p* = 0.11) and prostate cancer (*I*^2^ = 0%, *p* = 0.72) subgroups, but a significant heterogeneity was found in the melanoma (*I*^2^ = 73%, *p* < 0.01) and urothelial cancer (*I*^2^ = 61%, *p* = 0.11) subgroups.

**Table 2:** The summary of OS and PFS heterogeneity test.

Subgroup	OS		PFS	
	I2	p	I2	p
**Melanoma**	38.00%	0.16	73.00%	<0.01
**SCLC**	0.00%	0.83	28.00%	0.24
**NSCLC**	0.00%	0.48	45.00%	0.11
**Prostate**	77%	0.04	0.00%	0.72
**Urothelial**	0.00%	0.38	61.00%	0.11
**Total**	0.41	0.02	0.58	<0.01

### Publication Bias Analysis and Sensitivity Analysis

**Figure 2:**
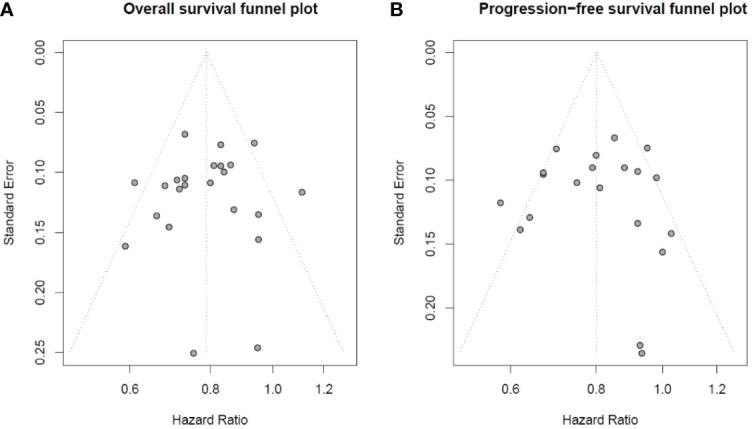
Begg’s funnel plot of overall survival and progression-free survival studies: **(A)** Begg’s funnel plot of overall survival studies to evaluate publication bias. **(B)** Begg’s funnel plot of progression-free survival studies to evaluate publication bias.

The *p*-values of Begg’s test and Egger’s test were applied for OS and PFS. We did not find publication bias in OS by Begg’s test (*p* = 0.5436) and Egger’s test (*p* = 0.6849), and in PFS by Begg’s test (*p* = 0.9483) and Egger’s test (*p* = 0.9774). The result of the OS and PFS publication bias analysis is directly reflected in **Figure 2** by using Begg’s funnel plot.

### Association of ICIs With Overall Survival

**Figure 3:**
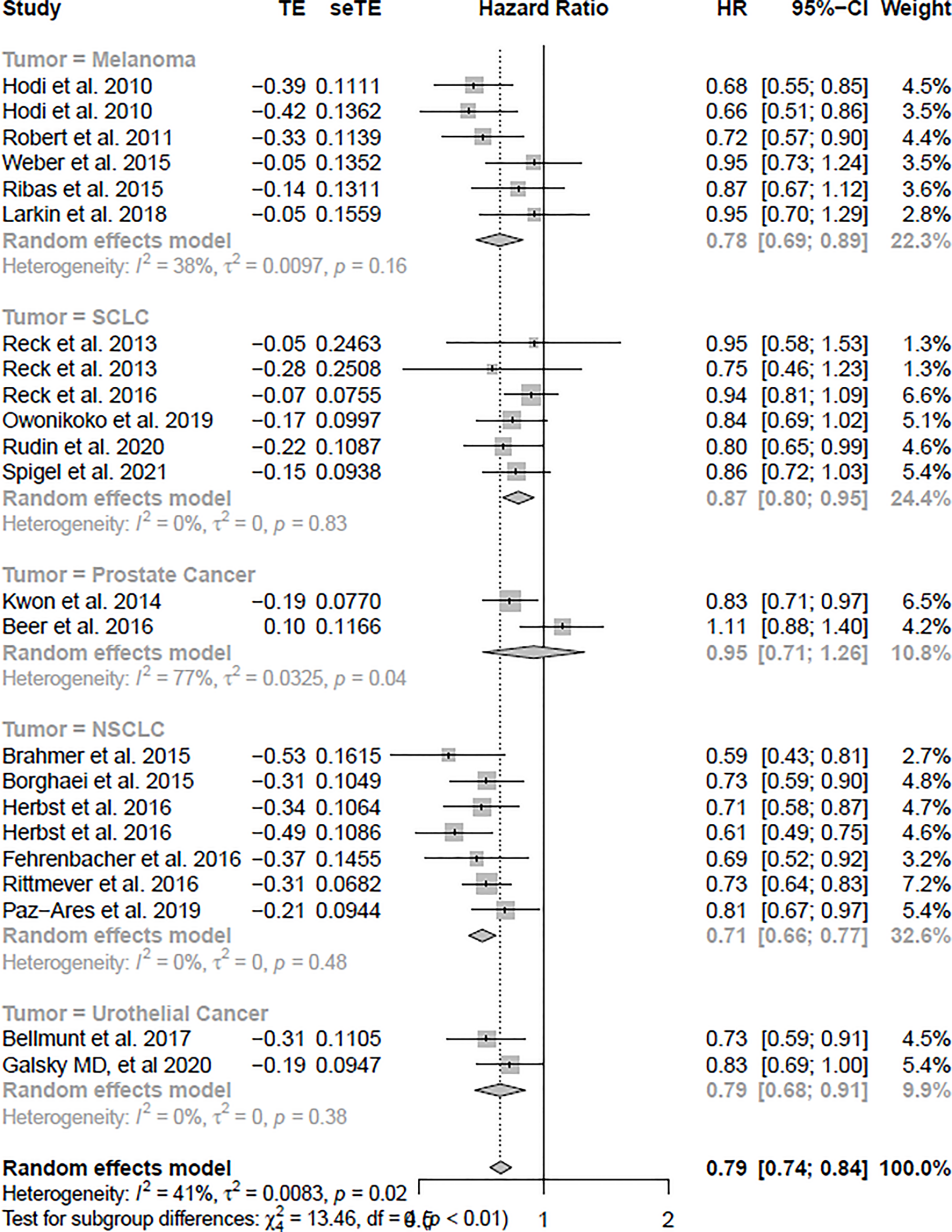
The forest plot of OS in the random-effect model.

**Figure 4:**
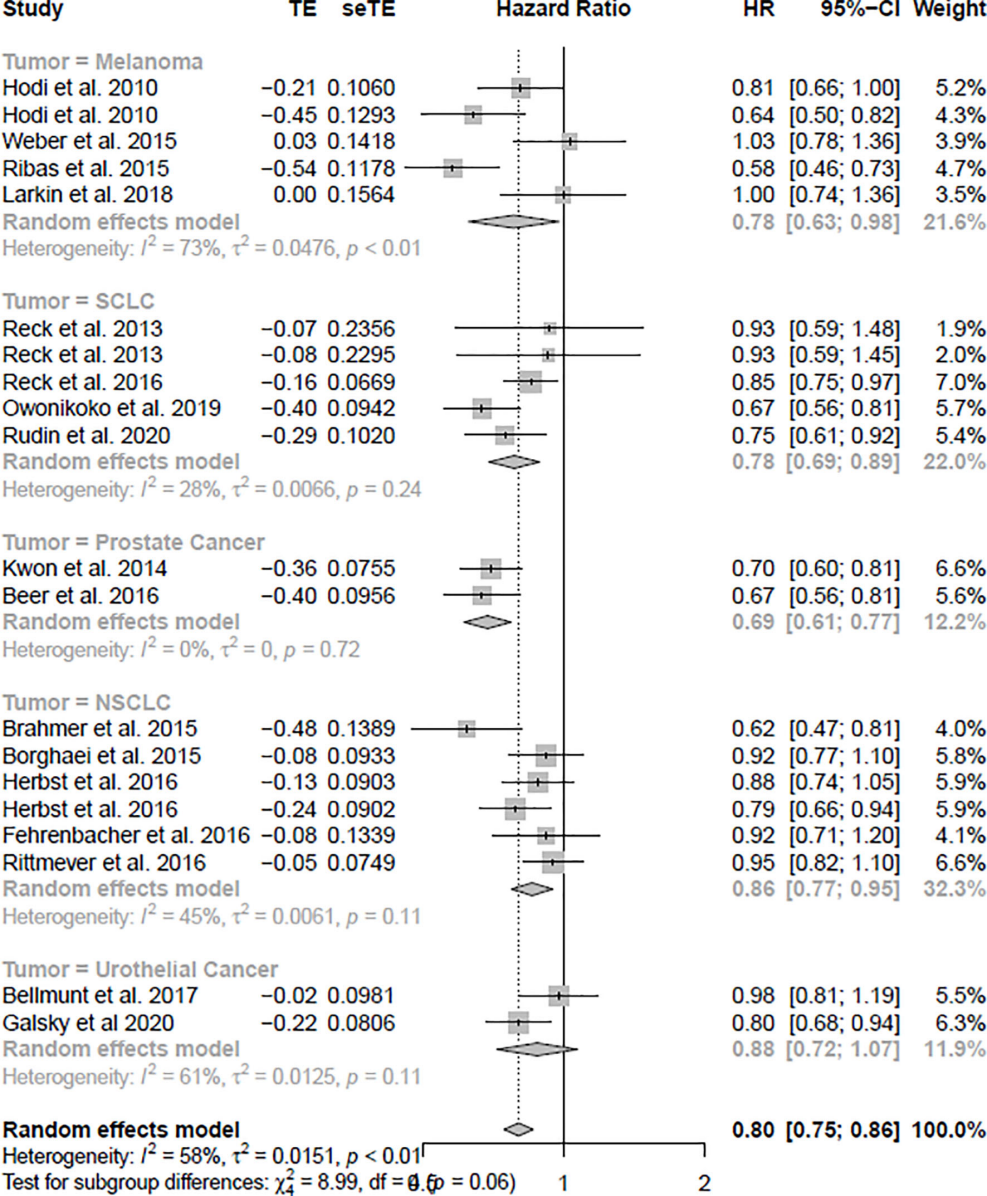
The forest plot of PFS in the random effect model.

The OS analysis was included in 23 studies, and the PFS analysis was included in 20 studies (shown in **Table 1**). **Figure 3** shows the results of OS, and **Figure 4** shows the results of PFS. **Table 3** shows the summary of the melanoma, SCLC, NSCLC, prostate cancer, and urothelial cancer (*I^2^* = 0%, *p* = 0.73) subgroups, but a significant heterogeneity was found in the prostate cancer subgroup meta-analysis and overall meta-analysis.

**Table 3:** The summary of the meta-analysis with OS and PFS.

Subgroup	OS		PFS	
	HR	95% CI	HR	95% CI
**Melanoma**	0.78	0.69–0.89	0.78	0.63–0.98
**SCLC**	0.87	080–0.95	0.78	0.69–0.89
**NSCLC**	0.71	0.66–0.77	0.86	0.77–0.95
**Prostate**	0.95	0.71–1.26	0.69	0.61–0.77
**Urothelial**	0.79	0.68–0.91	0.88	0.72–1.07
**Total**	0.79	0.74–0.84	0.80	0.75–0.86

In the OS analysis, the ICIs were associated with substantially ameliorated OS (HR = 0.79, CI = 0.74–0.84), compared with non-ICI therapies. In the subgroup analyses, melanoma, SCLC, NSCLC, and urothelial cancer patients treated with ICIs were associated more with OS compared with non-ICI therapies (HR = 0.78, CI = 0.69–0.89; HR = 0.87, CI = 0.80–0.95; HR = 0.71, CI = 0.66–0.77; HR = 0.79, CI = 0.68–0.91), respectively. However, prostate cancer was not significantly associated with improved OS (HR = 0.95, CI = 0.71–1.26).

### Association of ICIs With Progression-Free Survival

In the PFS analysis, the ICIs were associated with significantly improved PFS (HR = 0.80, CI = 0.75–0.86), compared with non-ICI therapies. In subgroup analyses, melanoma, SCLC, NSCLC, and prostate cancer patients treated with ICIs were associated more with PFS compared with non-ICI therapies (HR = 0.78, CI = 0.63–0.98; HR = 0.78, CI = 0.69–0.89; HR = 0.86, CI = 0.77–0.95; HR = 0.69, CI = 0.61–0.77), respectively. However, urothelial cancer was not significantly associated with improved PFS (HR = 0.88, CI = 0.72–1.05).

## Discussion

In our meta-analysis, a total of 12,126 participants (6,450 cases and 5,676 controls), treated with ICIs and non-ICI arms, from 20 articles were included.

In total, among 12,126 patients in our meta-analysis, 2,423 patients (1,514 cases and 969 controls) were included into the melanoma subgroup, 2,727 patients (1,353 cases and 1,374 controls) were included into the SCLC subgroup, 4,122 patients (2,063 cases and 2,059 controls) were included into the NSCLC subgroup, 1,401 patients (799 cases and 602 controls) were included into the prostate subgroup, and 1,393 patients (721 cases and 672 controls) were included into the urothelial cancer subgroup.

To our knowledge, this is the comprehensive meta-analysis to assess the efficacy of ICIs (ipilimumab, pembrolizumab, nivolumab, and atezolizumab) in different types of tumors, including melanoma, SCLC, NSCLC, prostate cancer, and urothelial cancer. Results of trials on ICIs have been published, while the clinical value of ICIs is still controversial. To further investigate the efficacy of ICIs, we made five subgroups of melanoma, SCLC, NSCLC, prostate cancer, and urothelial cancer with OS and PFS.

The pooled analyses indicated that ICIs were associated with obviously ameliorated PFS and OS compared with non-ICI arms. In OS subgroup analyses, melanoma, SCLC, NSCLC, and urothelial cancer patients treated with ICIs were associated more with OS compared with non-ICI therapies. However, prostate cancer was not significantly associated with improved OS. In PFS subgroup analyses, melanoma, SCLC, NSCLC, and prostate cancer patients treated with ICIs were associated more with PFS compared with non-ICI therapies. However, urothelial cancer was not significantly associated with improved PFS.

However, the meta-analysis had some limitations. To begin with, the number of participants in our meta-analysis was 12,126, and more studies should be added to this meta-analysis. Second, some heterogeneity existed in this meta-analysis, especially in the PFS group. It should be solved in the further study. Besides, more studies should be added into the prostate cancer patients and urothelial cancer subgroups.

## Conclusions

This meta-analysis got a conclusion that immune checkpoint inhibitors were associated with obviously ameliorated PFS and OS compared with non-ICI therapies.

## Data Availability Statement

The original contributions presented in the study are included in the article/supplementary material. Further inquiries can be directed to the corresponding author.

## Ethics Statement

Ethical review and approval were not required for the study on human participants in accordance with the local legislation and institutional requirements. Written informed consent for participation was not required for this study in accordance with the national legislation and the institutional requirements.

## Author Contributions

WD and LX wrote the manuscript. WD and XY collected the data and conducted the experiment. LX performed the project. WD interpreted the results. WD and LX developed the analytical tools. All authors contributed to the article and approved the submitted version.

## Funding

This research was funded in part by the National Natural Science Foundation of China, grant number 62172122, and the Fundamental Research Funds for the Central Universities, Jilin University, grant number 93K172021K04.

## Conflict of Interest

The authors declare that the research was conducted in the absence of any commercial or financial relationships that could be construed as a potential conflict of interest.

The reviewer XL declared a shared affiliation with the author XL to the handling editor at the time of review.

## Publisher’s Note

All claims expressed in this article are solely those of the authors and do not necessarily represent those of their affiliated organizations, or those of the publisher, the editors and the reviewers. Any product that may be evaluated in this article, or claim that may be made by its manufacturer, is not guaranteed or endorsed by the publisher.
